# Intrinsic Defects in B Cell Development and Differentiation, T Cell Exhaustion and Altered Unconventional T Cell Generation Characterize Human Adenosine Deaminase Type 2 Deficiency

**DOI:** 10.1007/s10875-021-01141-0

**Published:** 2021-10-17

**Authors:** Jin Yan Yap, Leen Moens, Ming-Wei Lin, Alisa Kane, Anthony Kelleher, Catherine Toong, Kathy H.C. Wu, William A. Sewell, Tri Giang Phan, Georgina E. Hollway, Karen Enthoven, Paul E. Gray, Jose Casas-Martin, Carine Wouters, Lien De Somer, Michael Hershfield, Giorgia Bucciol, Selket Delafontaine, Cindy S. Ma, Stuart G. Tangye, Isabelle Meyts

**Affiliations:** 1 Present Address: Garvan Institute of Medical Research, Darlinghurst, NSW Australia; 2 Clinical Immunogenomics Research Consortium of Australasia (CIRCA), Sydney, NSW Australia; 3 Department of Microbiology, Immunology and Transplantation, Laboratory for Inborn Errors of Immunity, KU Leuven, Herestraat 49, 3000 Leuven, EU Belgium; 4 Department of Clinical Immunology and Immunopathology, Westmead Hospital, Westmead, NSW Australia; 5 Faculty of Medicine, University of Sydney, Sydney, Australia; 6 Department of Immunology, Liverpool Hospital, Allergy and HIV, Liverpool, Sydney Australia; 7 HIV and Immunology Unit, St Vincent’s Hospital, Darlinghurst, NSW Australia; 8 Faculty of Medicine, St Vincent’s Clinical School, Sydney, NSW Australia; 9 The Kirby Institute for Infection and Immunity in Society, Sydney, Australia; 10 St Vincent’s Clinical Genomics, St Vincent’s Hospital Darlinghurst, Darlinghurst, NSW Australia; 11 School of Medicine, UNSW Sydney, Sydney, Australia; 12 Discipline of Genetic Medicine, University of Sydney, Sydney, Australia; 13 School of Medicine, University of Notre Dame, Fremantle, Australia; 14 Department of Immunology and Infectious Diseases, Sydney Children’s Hospital, Sydney, Australia; 15 School of Women’s and Children’s Health, UNSW Sydney, Sydney, NSW Australia; 16 Department of Microbiology and Immunology, Herestraat 49, 3000 Leuven, EU Belgium; 17 Department of Pediatrics, University Hospitals Leuven, Leuven, EU Belgium; 18 Department of Medicine and Biochemistry, Duke University Medical Center, Durham, NC USA; 19 Department of Pediatrics, Division of Inborn Errors of Immunity, University Hospitals Leuven, Herestraat 49, 3000 Leuven, EU Leuven Belgium

**Keywords:** ADA2 deficiency, DADA2, T cell exhaustion, Humoral immunodeficiency, SIGLEC-1, Type I IFN signature, Monocytes

## Abstract

**Purpose:**

Deficiency of adenosine deaminase type 2 (ADA2) (DADA2) is a rare inborn error of immunity caused by deleterious biallelic mutations in *ADA2*. Clinical manifestations are diverse, ranging from severe vasculopathy with lacunar strokes to immunodeficiency with viral infections, hypogammaglobulinemia and bone marrow failure. Limited data are available on the phenotype and function of leukocytes from DADA2 patients. The aim of this study was to perform in-depth immunophenotyping and functional analysis of the impact of DADA2 on human lymphocytes.

**Methods:**

In-depth immunophenotyping and functional analyses were performed on ten patients with confirmed DADA2 and compared to heterozygous carriers of pathogenic *ADA2* mutations and normal healthy controls.

**Results:**

The median age of the patients was 10 years (mean 20.7 years, range 1–44 years). Four out of ten patients were on treatment with steroids and/or etanercept or other immunosuppressives. We confirmed a defect in terminal B cell differentiation in DADA2 and reveal a block in B cell development in the bone marrow at the pro-B to pre-B cell stage. We also show impaired differentiation of CD4^+^ and CD8^+^ memory T cells, accelerated exhaustion/senescence, and impaired survival and granzyme production by ADA2 deficient CD8^+^ T cells. Unconventional T cells (i.e. iNKT, MAIT, Vδ2^+^ γδT) were diminished whereas pro-inflammatory monocytes and CD56^brigh^^t^ immature NK cells were increased. Expression of the IFN-induced lectin SIGLEC1 was increased on all monocyte subsets in DADA2 patients compared to healthy donors. Interestingly, the phenotype and function of lymphocytes from healthy heterozygous carriers were often intermediate to that of healthy donors and ADA2-deficient patients.

**Conclusion:**

Extended immunophenotyping in DADA2 patients shows a complex immunophenotype. Our findings provide insight into the cellular mechanisms underlying some of the complex and heterogenous clinical features of DADA2. More research is needed to design targeted therapy to prevent viral infections in these patients with excessive inflammation as the overarching phenotype.

**Supplementary Information:**

The online version contains supplementary material available at 10.1007/s10875-021-01141-0.

## Introduction

In 2014, biallelic loss-of-function mutations in *ADA2* (encoding adenosine deaminase type 2 [ADA2]) were described as the cause of a novel rare inborn error of immunity [1, 2]. The clinical phenotype of human ADA2 deficiency includes not only recurrent fevers and vasculitis (ranging from livedo racemosa to polyarteritis nodosa and lacunar stroke) but also immunodeficiency and cytopenia, either due to autoimmunity or bone marrow (BM) failure and hematological malignancy [3–5]. The pathophysiology of deficiency of ADA2 (DADA2) is far from resolved. Disrupted interaction between the vascular endothelium and monocytes is believed to be central to at least the vascular phenotype of the disease, with a skewing of macrophages to a pro-inflammatory M1 phenotype [1]. For patients with a predominant vasculitis phenotype, the mainstay of treatment is TNF inhibition [6]. However, cytopenias are mostly refractory to treatment with TNF inhibitors, growth factors and immunosuppressives [7, 8]. Hematopoietic stem cell transplantation results in excellent survival and cures ADA2-deficient patients from all disease manifestations, demonstrating the hematopoietic-intrinsic etiology of this condition [9, 10]. In terms of immunodeficiency, up to 30% of DADA2 patients have panhypogammaglobulinemia (IgG, IgM, IgA) [3] and can present with a common variable immunodeficiency-like phenotype, including recurrent upper and lower respiratory tract infections, bronchiectasis and associated gastrointestinal involvement. However, some patients also exhibit a phenotype mimicking auto-immune lymphoproliferative syndrome (ALPS) [11–13]. Moreover, DADA2 patients can have particular susceptibility to herpes virus infections—cytomegalovirus (CMV), Epstein Barr virus (EBV), herpes simplex virus 1, human herpes virus 6 (HHV6)—as well as HPV and *Molluscum contagiosum* [12, 14].

While features of vasculitis in DADA2 are well described, mechanisms underlying immunodeficiency have been less well explored. Initial descriptions noted B cell lymphopenia, progressive hypogammaglobulinemia occasionally mimicking agammaglobulinemia, increased numbers of naïve and CD21^lo^ B cells, but reductions in switched memory B cells and plasmablasts [1, 15, 16]. In vitro findings of decreased T-dependent Ig secretion and increased B cell apoptosis inferred that a B cell–intrinsic defect underlies humoral immunodeficiency in DADA2 [1, 16]. However, while circulating T follicular helper (cTfh) cells—the CD4^+^ T cell subset that guides B cell differentiation [17]—have been reported to be present in normal or increased proportions in DADA2 patients, ADA2-deficient CD4^+^ T cells apparently exhibit features of impaired help for B cell differentiation such as reduced IL-21 production and CD40L expression in vitro [16]. Thus, functionally impaired cTfh cells may also contribute to the B cell defect in DADA2. Further cellular aberrations in DADA2 include increased proportions of CD4^−^CD8^−^ αß^+^ TCR^+^ cells [1, 13, 18, 19] consistent with an ALPS-like phenotype as well as reductions in frequencies of central and effector memory CD4^+^ and CD8^+^ T cell subsets [1, 13, 16]. In order to further delineate peripheral immune defects in DADA2, we conducted an in-depth clinical, immunophenotyping and functional study in 10 patients with bi-allelic pathogenic *ADA2* variants from 6 unrelated families.

## Material and Methods

### Patients

Patients with DADA2 were recruited from the University Hospitals Leuven Primary Immunodeficiency Clinic, Leuven, Belgium, and from St Vincent’s, Liverpool and Westmead Hospitals in Sydney, Australia. Healthy controls were recruited from volunteers, or purchased as donor buffy coats from the Australian Red Cross Blood Service. The study was approved by the respective ethics review boards of the participating institutes, including the ethics committees of University Hospitals Leuven (S63077, S63807 and S54866), Sydney Local Health District RPAH Zone Human Research Ethics Committee and Research Governance Office, Royal Prince Alfred Hospital, Camperdown, NSW, Australia (Protocol X16-0210/LNR/16/RPAH/257); the South East Sydney Local Health District Human Research Ethics Committee, Prince of Wales/Sydney Children’s Hospital, Randwick, NSW, Australia (Protocol HREC/11/POWH/152). Written informed consent for genetic investigations and immunological analyses, as well as the publication of data, was obtained from each family. DADA2 was diagnosed based on the demonstration of biallelic deleterious mutations in *ADA2*, in association with deficient plasma ADA2 enzyme activity.

### Detailed Immunophenotyping and Functional Analysis of ADA2-Deficient Lymphocytes

PBMCs from ADA2-deficient patients (*n* = 10), heterozygous healthy carriers (*n* = 5) and healthy donors (*n* = 27) were analyzed as described previously to determine proportions of B cells (CD20^+^), T cells (CD3^+^), NK cells (CD3^−^CD56^+^) and dendritic cells (DCs) (CD19^−^CD3^−^CD56^−^CD235a^−^CD14^−^CD20^−^ HLA-DR^+^) [20]. Subsets of T cells (CD4^+^, CD8^+^, iNKT [TCR Vα24Vβ11^+^], MAIT [TCR Vα7.2^+^CD161+], Vγδ [TCR Vγδ^+^], Vαβ [TCR Vαβ^+^]) were also determined [20]. Frequencies of naïve (CD45RA^+^CCR7^+^), central memory (T_CM_, CCR7^+^CD45RA^−^), effector memory (T_EM_, CCR7^−^CD45RA^−^) and revertant memory T_EMRA_, CD45RA^+^CCR7^−^) cells within the CD4^+^ and CD8^+^ T-cell populations and of Treg (CD4^+^CD25^hi^CD127^−^) and Tfh cells (CD4^+^CD45RA^−^CXCR5^+^) were enumerated as percentages of total CD4^+^ or CD8^+^ T cells. Th1, Th2, Th17 and Th1/Th17 (Th1*)-like phenotypes within memory CD4^+^ and Tfh cells were defined by differential expression of CXCR3 and CCR6 [17, 20–22]. Frequencies of transitional (CD27^−^CD10^+^), naïve (CD27^−^CD10^−^), total memory (CD10^−^CD27^+^), class-switched memory (IgD^−^IgM^−^, IgG^+^ or IgA^+^) and CD21^low^ (CD19^+^CD21^−^) CD20^+^ B cells were also determined. Subsets of total NK cells were defined: immature CD56^bright^, mature CD56^dim^CD57- and terminally differentiated CD56^dim^CD57^+^ cells. Classical (CD14^+^CD16^−^), intermediate (CD14^+^CD16^+^) and non-classical (CD14^−^CD16^+^) subsets of monocytes were analyzed [23]. Lastly, DCs were assessed by quantifying proportions of plasmacytoid DCs (CD123^+^CD11c^−^), myeloid DCs (mDCs, CD11c^+^CD123) and mDC subsets: DC1 (CD141^+^ mDCs, CD16^+^ mDCs, CD1c^+^ mDCs and CD1c^lo/−^. BM aspirates were incubated with mAbs against CD34, CD19, CD20, CD10, IgM and IgD. Populations of B-lineage cells (CD19^+^), as well as pro-B (CD19^+^CD34^+^CD10^hi^CD20^−^IgM^−^), pre-BI (CD19^+^CD34^−^CD10^hi^CD20^−^IgM^−^), pre-BII (CD19^+^CD34^−^CD10^hi^CD20^dim^IgM^−/+^), immature (CD19^+^CD34^−^CD10^int^CD20^+^IgM^+^) and recirculating mature (CD19^+^CD34^−^CD10^−^CD20^+^) B cells, were quantified [24–26].

### Functional Analysis of ADA2-Deficient Lymphocytes

Naive and memory CD4^+^ T cells were isolated after excluding Tregs (CD25^hi^CD127^lo^) and sorting CD4^+^CD45RA^+^CCR7^+^ and CD4^+^CD45RA^−^ cells respectively. Total CD8^+^ T and naive B cells were isolated as CD8^+^CD4^−^ and CD20^+^CD10^−^CD27^−^IgG^−^ cells, respectively. Sorted lymphocyte populations were labeled with the division-tracing dye CellTrace™ Yellow (CTY). CD4^+^ T cells were cultured under Th0, or Th1-, or Th17-polarizing conditions and cytokine expression and production were determined [27]. B cells were cultured with CD40L (200 ng/ml) alone or in the absence or presence of IL-21 (50 ng/ml). Cells were harvested, stained with Zombie dye to determine viability and enumerated with calibrate beads. Ig secretion was determined by ELISA as described [28]. CD8^+^ T cells were cultured with T cell expansion and activation (TAE; anti-CD3, anti-CD28, anti-CD2 mAb) beads (Miltenyi Biotech, Bergisch Gladbach, Germany). After 4 days of culture, supernatants were harvested and assayed for production of interferon (IFN)γ, tumor necrosis factor (TNF)-α, interleukin (IL)-2 and Granzyme A and Granzyme B by cytometric bead array (Becton Dickinson) [29]. For all cultures, cell viability was determined by Zombie Aqua™ live/dead discrimination dye, and proliferation was assessed by the dilution of CTY.

### ADA2 Testing

For all patients, plasma ADA2 enzyme activity was measured by HPLC [30] and spectrophotometric [31] assays, as described previously. The ADA2 activity in extracts of dried plasma spots was measured as described [32].

### IFN Score

The expression of six interferon-stimulated genes (ISGs) was measured by quantitative PCR, and the median fold change was used to calculate an interferon score (IS) for each subject compared to a previously derived panel of 20 or 13 local controls, respectively, in the Leuven and Sydney sites (where a reading + 2 SDs above the mean of healthy donors, i.e. an IS of > 2.466 or > 2.188 for Leuven and Sydney respectively is considered abnormal) as previously described [33].

### Statistical Analysis

Significant differences were determined by multiple *t*-tests with *P* < 0.05 indicating statistical significance or by Mann–Whitney *U* tests (GraphPad Prism v. 8.4.2, La Jolla, CA, USA). Differences in mean values were considered significant at *P* < 0.05.

## Results

### Clinical Characteristics, ADA2 Mutations and Plasma Enzyme Activity of ADA2-Deficient Patients

Ten DADA2 patients (age range: 1 year and 10 months to 44 years) from 6 unrelated families, 5 carriers (from 3 families) (age range: 43–73 years) and 27 healthy controls (age range: 18–65 years) were included. Details on the clinical phenotype, *ADA2* genotype and ADA2 enzymatic activity of patients, heterozygous carriers and healthy donors are listed in Table 1. Assessment of ADA2 function confirmed that all patients with bi-allelic *ADA2* variants had dramatically reduced to undetectable levels of serum ADA2 activity, while all heterozygous carriers exhibited detectable ADA2 levels, albeit reduced compared to healthy donors.

**Table 1:** Genetic diagnosis, ADA2 enzyme activity and clinical data of ADA2-deficient patients

	Kindred	Genetic diagnosis	ADA2 level mU/g protein	Age at sample	Viral phenotype	Treatment at sampling	Hypo-gamma	Type I IFN score	Major clinical phenotype at sampling	Other findings
1	A	c.973-2 T > C / c. del1240-1442	1.1	8y6m	Warts, mollusca	(-) SCIG, amoxicilline	y	19	V (livedo), W, N, T, AIHA,	Severe AE to CPV
2	A	c.973-2 T > C / c. del1240-1442	2.7	10y8m	Warts, mollusca	(-)	y	7	V (livedo), R,	
3	B	c.140G > T (p.Gly47Val)/ c.506G > A(p.Arg169Gln)	3.3	1y10m		(-)	n	4	None; now V (livedo), CNS, N,	
4	B	c.140G > T (p.Gly47Val)/ c.506G > A(p.Arg169Gln)	2.1	4y8m		Etanercept	y	22,5	V (livedo, cutaneous papules), now N	
5	C	c.973-2 T > C / c.973-2 T > C	1.6	5y8m		Etanerecept	n	5	V (livedo	
6	D	p.Gly47Arg / p.Gly47Arg	1.9	11y11m		Tocilizumab	n	3	Castleman disease V (livedo),	Later: CNS microbleeds, testicular thrombosis
7	E	c.506G > A, c.1358A > G	2.6 (2.6 HPLC)	33y5m	Recurrent VZV, warts	Prednisone 7.5 mg daily, IVIG (previously received mtx, cpm, mmf)	y	4.3*	DVT at 10y, V intestinal, S, CNS at 16y, HT	AE to UPV, Candida infections esophagus, skin, nails
8	E	c.506G > A, c.1358A > G	0 (2.9 HPLC)	40y	Viral encephalitis, Shingles	(-)	y	6.7*	CNS at 3,5y, V, HT	AE to UPV
9	E	c.506G > A, c.1358A > G	2.1 HPLC	44y	Shingles, EBV	(-)	y (IgM)	6.95*	V	AE to UPV
10	F	c.1397_1403del;c.1397_1403del p.Lys466Thrfs*2/p.Lys466Thrfs*2	0 (2.9 HPLC)	42y	(-) recent EBV and HSV1, not at sampling	SCIG	y	15.5*	P, HSM, A	Hepatitis (not at sampling)

*A* Alopecia; *AE* adverse event; *AIHA* auto-immune hemolytic anemia; *CNS* central nervous system vasculitis manifestation, lacunar infarction, cranial nerve palsy, stroke, hemorrhage; *cpm* cyclophosphamide; *CPV* conjugated pneumococcal vaccine; *DVT* deep venous thrombosis; *HG* hypogammaglobulinemia; *HSM* hepatosplenomegaly; *HT* hypertension; *IVIG* intravenous immunoglobulins; *mtx* methotrexate; *mmf* mofetil mycophenolate; *N* neutropenia; *P* pancytopenia; *R* Raynaud phenomenon; *S* splenomegaly; *SCIG* subcutaneous immunoglobulins; *T* thrombocytopenia; *UPV* unconjugated pneumococcal vaccine; *V* vasculitis

^*^IFN score analyses performed in Sydney site

All patients suffered from vasculitis, ranging from livedo racemosa to CNS vasculopathy (transient ischemic attack to lacunar infarction). Severe viral infections were present in five out of ten patients ranging from warts to viral encephalitis. Six of the 10 patients had hypogammaglobulinemia. Interestingly, two of ten patients experienced severe local reactions as well as fever and general malaise upon vaccination with unconjugated pneumococcal vaccine. Another patient experienced fever malaise upon vaccination with conjugated pneumococcal vaccine and live attenuated MMR vaccine after which vasculitis occurred for the first time. Overall, there was significant phenotypic heterogeneity in the severity of vasculitis and spectrum of organ involvement within individual kindreds.

### Human B Cell Development and Differentiation Is Compromised by ADA2 Deficiency

**Fig. 1:**
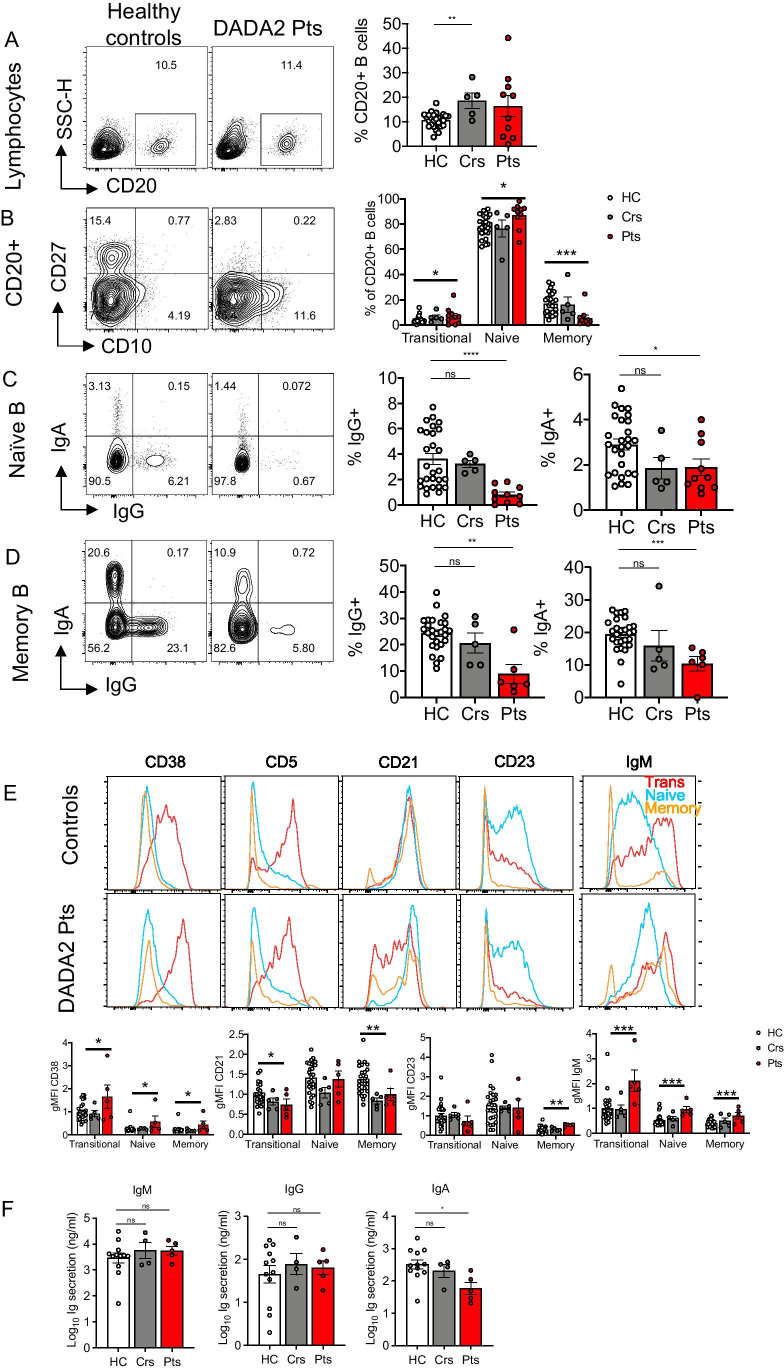
Impairment in peripheral B cell development and differentiation in DADA2 patients. Immunophenotyping was determined by flow cytometry on PBMCs from healthy controls (HC), heterozygous carriers (Crs) and DADA2 patients (Pts). Flow cytometric plots and graphs showing the frequencies of **A** CD20^+^ B cells within the lymphocyte population; **B** transitional (CD10^+^CD27^−^), naïve (CD10^−^CD27^−^) and memory (CD10^−^CD27^+^) subsets within the CD20^+^ B cell population; **C, D** IgA^+^ and IgG^+^ cells within the (C) CD27^−^ and (D) CD27^+^ memory B cell populations; **E** Representative histogram plots (top panels) show expression of CD38, CD21, CD23, and IgM on transitional (red), naïve (blue) and memory (orange) B cells of healthy donors and DADA2 patients, and summarized in graphs (bottom panels) depicting geometric mean fluorescent intensity (gMFI) normalized to the transitional B cell population of healthy controls. **F** Sort-purified naïve B cells from healthy controls (HC), heterozygous carriers (Crs) and DADA2 patients (Pts) were stimulated in vitro with CD40L/IL-21 for 7 days. IgM, IgG and IgA secretion was determined by ELISA. Graphs represent the mean ± S.E.M.; each symbol represents an individual. Significant differences were determined by multiple *t-*tests with *P* < 0.05 indicating statistical significance or by Mann–Whitney *t*-tests with *P < 0.05; **P < 0.01; ***P < 0.001

Flow cytometric analysis revealed comparable frequencies of CD20^+^ B cells in peripheral blood of DADA2 patients (mean = 16.4%) and healthy controls (10.6%; Fig. 1A). Delineation of B cell subsets revealed significant increases in percentages of transitional and naïve B cells in DADA2 patients compared to healthy controls. Consistent with previous studies [1, 12, 13, 16], memory B cells were significantly reduced in DADA2 patients compared to healthy donors (Fig. 1B).

We next assessed Ig class switching in vivo by quantifying the frequencies of B cells that expressed IgG or IgA. In healthy donors, the vast majority of class-switched B cells are contained within the CD27^+^ memory B cell subset; however, a small proportion of IgG^+^ and IgA^+^ B cells can also be detected within the CD27^−^ B cell subset [34]. Analysis of these B cell subsets demonstrated significantly decreased proportions (~ fivefold) of IgG^+^ and IgA^+^ B cells in both the CD27^−^ (naïve) (Fig. 1C) and CD27^+^ (memory) (Fig. 1D) B cell compartments. While frequencies of total B cells were increased in healthy heterozygous carriers (Fig. 1A), proportions of transitional, naïve, memory B cells (Fig. 1B) and class-switched B cells (Fig. 1C, D) in heterozygous carriers were similar to healthy donors. Together, these findings indicate that ADA2-deficiency compromises not only the generation and/or maintenance of the memory B cell pool, but also the ability of B cells to undergo Ig class switching.

### Circulating B Cells in ADA2-Deficient Patients Exhibit a Block in Maturation

The increase in transitional and decrease in total and class-switched memory B cells led us to further examine the maturation status of B cell subsets in DADA2 patients. This was achieved by assessing the expression of a range of surface molecules that are differentially expressed as human B cells mature from transitional to naïve, and naïve to memory B cells [24, 35, 36]. In healthy donors, CD38 and IgM are highly expressed on transitional B cells and then downregulated on naïve and memory B cells [24, 35, 36] (Fig. 1E). While this pattern was observed for ADA2-deficient B cell subsets (Fig. 1E), the absolute levels of CD38 and IgM expressed were significantly (~ twofold) higher on transitional, naïve and memory B cells from DADA2 patients than those from healthy donors (Fig. 1E).

CD21 and CD23 increase as transitional B cells from healthy donors develop into naïve B cells, and then plateau/decrease as naïve B cells differentiate into memory B cells [24, 36] (Fig. 1E). Compared to healthy donors, CD21 was consistently and significantly reduced on ADA2-deficient transitional B cells and failed to be upregulated on ADA2-deficient memory B cells (Fig. 1E). CD23 was also lower on ADA2-deficient transitional B cells (Fig. 1E). While CD23 was upregulated normally on ADA2-deficient naive B cells, it continued to be more highly expressed on ADA2-deficient memory B cells compared to memory B cells from healthy donors (Fig. 1E). No differences were observed for CD19 or BAFF-R expression on transitional, naïve or memory B cells from healthy donors and DADA2 patients (data not shown). The phenotype of transitional, naïve and memory B cells from healthy heterozygous carriers was comparable to that of healthy donors (Fig. 1E). Collectively, our detailed analysis of peripheral B cell subsets indicates that ADA2 deficiency compromises B cell differentiation, resulting in B cell populations that are developmentally less mature than those in healthy donors.

To further explore B cell–intrinsic defects due to ADA2-deficiency, we assessed the ability of naïve B cells to proliferate and differentiate in vitro in response to CD40L with or without IL-21, which mimics T-dependent B-cell activation [17, 37]. There were no significant differences in survival (Fig. S1A) or proliferation (Fig. S1B) of naïve B cells from DADA2 patients, heterozygous carriers or healthy donors 5 days post-stimulation. Similarly, induction of IgM and IgG secretion by CD40L/IL-21 stimulation was unaffected by heterozygous or bi-allelic mutations in *ADA2* (Fig. 1F). However, IgA secretion by naïve ADA2-deficient B cells was significantly reduced compared to healthy donor naïve B cells (Fig. 1F). Thus, naïve B cells in DADA2 patients have an intrinsically impaired ability to differentiate into IgA-secreting plasmablasts. Overall, ADA2 deficiency impacts differentiation of peripheral naive B cells to memory B cells, as well as class switching in vivo and generating of IgA-secreting cells in vitro.

### Arrested B Cell Development in ADA2-Deficient Bone Marrow

**Fig. 2:**
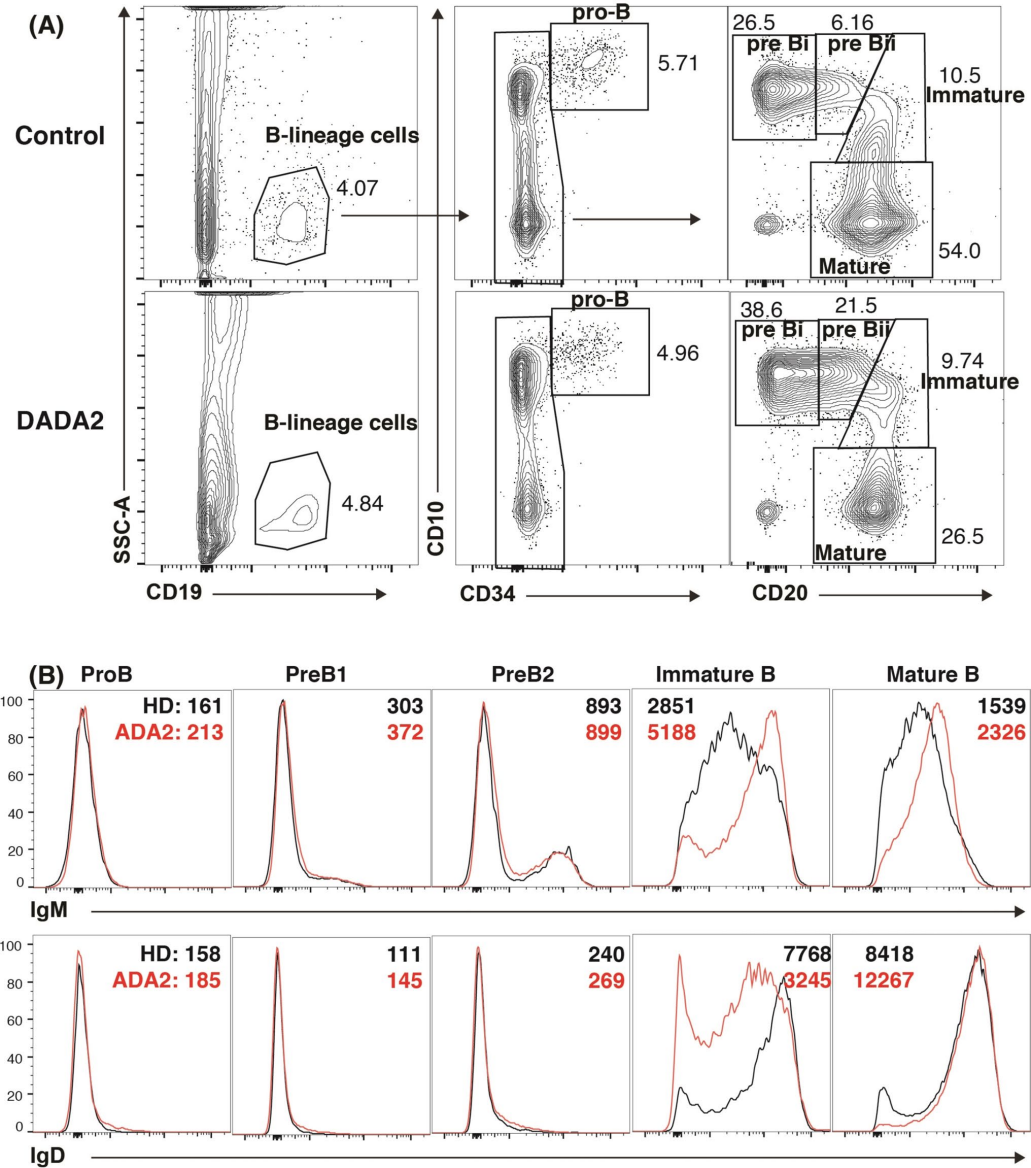
*ADA2* mutations impede B cell development in the bone marrow at the preB to immature B cell stage. **A**, **B** BM aspirates from healthy donors, or a DADA2 patient were labeled with mAbs against CD34, CD19, CD20, CD10, IgM and IgD. **A** Proportions of B-lineage cells (CD19^+^), pro-B (CD19^+^CD34^+^CD10^hi^), pre-BI (CD19^+^CD34^−^CD10^hi^), pre-BII (CD19^+^CD34^−^CD10^hi^), immature (CD19^+^CD34^−^CD10^int^) and recirculating mature (CD19^+^CD34^−^CD10^−^CD20^+^) B cells. **B** Each BM B cell subset in healthy donors (black histogram) and DADA2 patients (red histogram) was assessed for expression of IgM (upper) and IgD (lower panel). Values in **B** correspond to the gMFI of IgM and IgD expression on BM B cell subsets from in healthy donors (black) and the DADA2 patient (red)

To extend our observations into the defect in B cell development observed in peripheral blood, we quantified proportions of B-lineage cells (CD19^+^), as well as pro-B (CD19^+^CD34^+^CD10^hi^CD20^−^IgM^−^), pre-BI (CD19^+^CD34^−^CD10^hi^CD20^−^IgM^−^), pre-BII (CD19^+^CD34^−^CD10^hi^CD20^dim^IgM^−/+^), immature (CD19^+^CD34^−^CD10^int^CD20^+^IgM^+^) and mature (CD19^+^CD34^−^CD10^−^CD20^+^) B cells in the BM of a DADA2 patient who did not receive any immunosuppressive treatment or anti-TNF agent. Frequencies of total B-lineage cells and pro-B cells were comparable in the patient and healthy donors (Fig. 2A). However, pre-BI and pre-BII cells were markedly increased, and mature recirculating B cells reduced, in DADA2 BM compared to those in control BM (Fig. 2A). Although proportions of immature B cells were similar in the patient and controls, ADA2-deficient immature B cells exhibited features of altered maturation, evidenced by delayed down-regulation of CD10 (Fig. 2A), elevated expression of IgM (Fig. 2B), and reduced expression of IgD (Fig. 2B). Consistent with fewer class-switched memory B cells in the blood of DADA2 patients (Fig. 1C), > 98% of mature ADA2-deficient BM B cells were IgM^hi^IgD^+^, compared to ~ 80–85% in healthy controls (Fig. 2B). Thus, the aberrant distribution and phenotype of peripheral B cell subsets in ADA2-deficient patients likely result from a block in B cell development in the BM, evidenced by an accumulation of pre-B cells and underdeveloped immature B cells.

### CD4^+^ and CD8^+^ Memory T cells and Regulatory T Cells Are Reduced In Vivo in DADA2 Patients

**Fig. 3:**
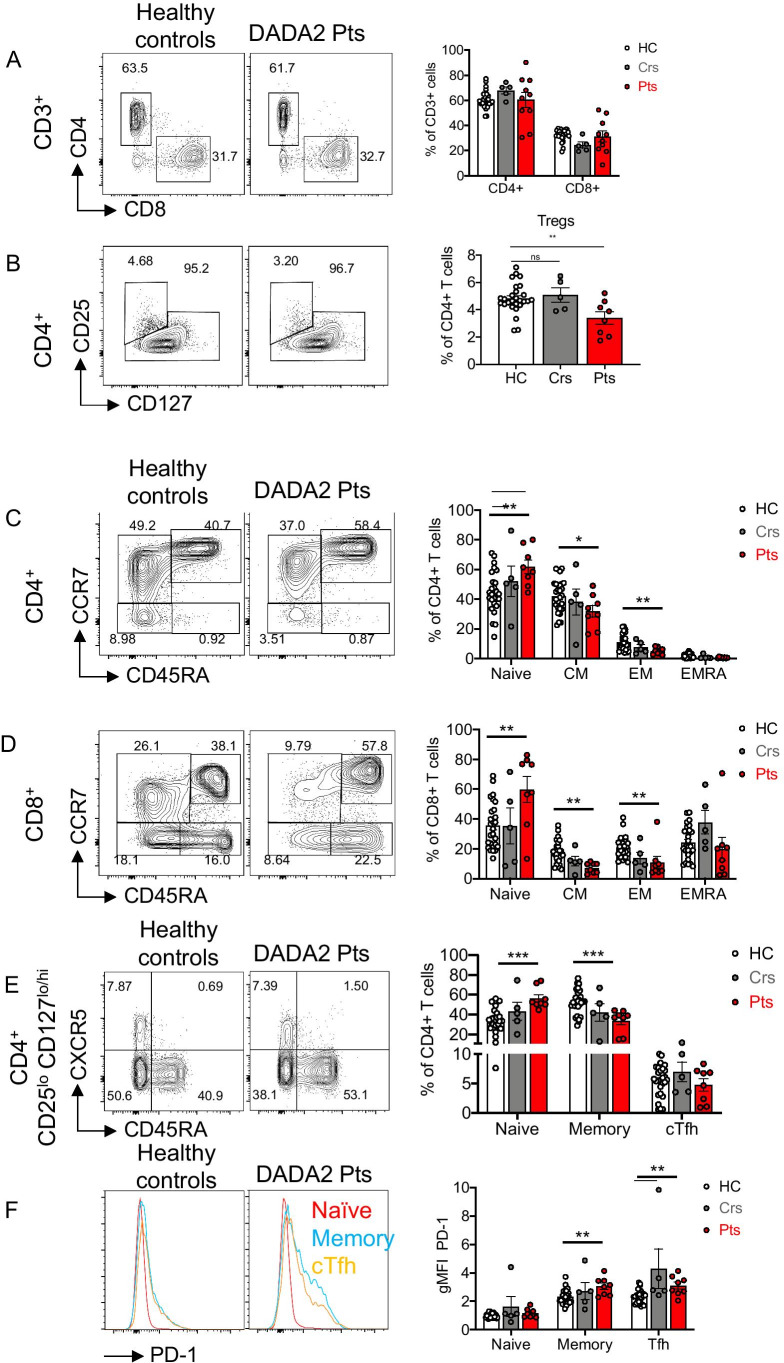
Aberrant CD4^+^ and CD8^+^ T cell differentiation in DADA2 patients. Immunophenotyping by flow cytometry on PBMCs from healthy controls (HC), heterozygous carriers (Crs) and DADA2 patients (Pts) was performed. Flow cytometric plots (left) and graphs (right) show the frequencies of **A** CD4^+^ and CD8^+^ T cells within the CD3^+^ lymphocyte population; **B** CD25^hi^CD127^lo^Treg within CD4^+^ T cells; **C, D** naïve (CD45RA^+^CCR7^+^), central memory (CM, CD45RA^−^CCR7^+^), effector memory (EM, CD45RA^−^CCR7^−^) and terminally differentiated effector memory (EMRA, CD45RA^+^CCR7^−^) subsets within **C** CD4^+^ T cells and **D** CD8^+^ T cells; **E** naïve (CD45RA^−^CXCR5^−^CCR7^+^), memory (CD45RA^−^CXCR5^−^) and cTfh (CD45RA^−^CXCR5^+^) within CD4^+^CD25^lo^CD127^lo/hI^ T cells. **F** Representative histogram plots (left) showing PD-1 expression on naïve (red), memory (blue) and cTfh (orange) CD4^+^ T cells from healthy controls and DADA2 patients, and summarized in graphs (right) as gMFI normalized to naïve CD4^+^ T cells of healthy controls. **A–F** Graphs represent the mean ± S.E.M.; each symbol represents an individual. Significant differences were determined by multiple *t*-tests with *P* < 0.05 indicating statistical significance or by Mann–Whitney *U*-tests with *P < 0.05; **P < 0.01; ***P < 0.001

We next examined the distribution and maturation status of T cells in DADA2 patients. Proportions of total (CD3^+^) T cells (Fig. S2A), as well as CD4^+^ and CD8^+^ T cells (Fig. 3A), in peripheral blood of DADA2 patients and healthy donors were comparable. Heterozygous carriers had modest but significantly increased CD4^+^ T cells and reduced CD8^+^ T cells (Fig. 3A). Within the population of CD4^+^ T cells, Treg cells (CD25^hi^CD127^lo^) were significantly reduced in DADA2 patients compared to those in healthy donors and heterozygous carriers (Fig. 3B). Frequencies of naïve (CD45RA^+^CCR7^+^) T cells were significantly increased, whereas central memory (T_CM_, CD45RA^−^CCR7^+^) and effector memory (T_EM_, CD45RA^−^CCR7^−^) T cells were significantly reduced within both the CD4^+^ and CD8^+^ T cell subsets in DADA2 (Fig. 3C, D). There were no differences in proportions of CD4^+^ or CD8^+^ T_EMRA_ (CD45RA^+^CCR7^−^) cell subsets. In general, the distribution of these CD4^+^ and CD8^+^ T cell subsets in healthy heterozygous carriers was comparable to healthy donors (Fig. 3C, D).

We also analyzed CD4^+^ T cells for cTfh cells based on differential expression of CD45RA, CXCR5 and PD-1 (CD45RA-CXCR5^+^). The cTfh cells were present in comparable frequencies in DADA2 patients, heterozygous healthy carriers and healthy donors (Fig. 3E). This was even more apparent when expressed as a proportion of memory CD4^+^ T cells (HC: 9.7 ± 1.1%; carriers: 16 ± 3.4%; ADA2: 15.5 ± 3.3%). The Tfh cells can also be characterized by assessing PD-1 expression, as well as delineation into distinct subsets defined by differential expression of the chemokine receptors CCR6 and CXCR3 [21, 38]. Consistent with our previous findings [21], PD-1 was expressed at low levels on naïve CD4^+^ T cells, and upregulated on memory CD4^+^ and cTfh cells from healthy donors (Fig. 3F). While similar expression patterns of PD-1 were observed for memory and cTfh cells from healthy heterozygous carriers, PD-1 was significantly increased on memory CD4^+^ T and cTfh cells from DADA2 patients (Fig. 3F). Proportions of Th1 (CXCR3^+^CCR6^−^), Th17 (CXCR3^−^CCR6^+^), Th2 (CXCR3^−^CCR6^−^) and Th1/Th17 (CXCR3^+^CCR6^+^)-type cells within both memory CD4^+^ T cells and cTfh cell populations were also comparable between DADA2 patients and healthy donors (Fig. S2B, C). Thus, although the generation of memory T cells is compromised of *ADA2* mutations, differentiation of memory CD4^+^ T cells to distinct effector fates remains intact. Consequently, there is a quantitative rather than qualitative effect of ADA2-deficiency on memory CD4^+^ T cells.

### CD4^+^ and CD8^+^ T Cell Differentiation Is Compromised by ADA2 Deficiency

**Fig. 4:**
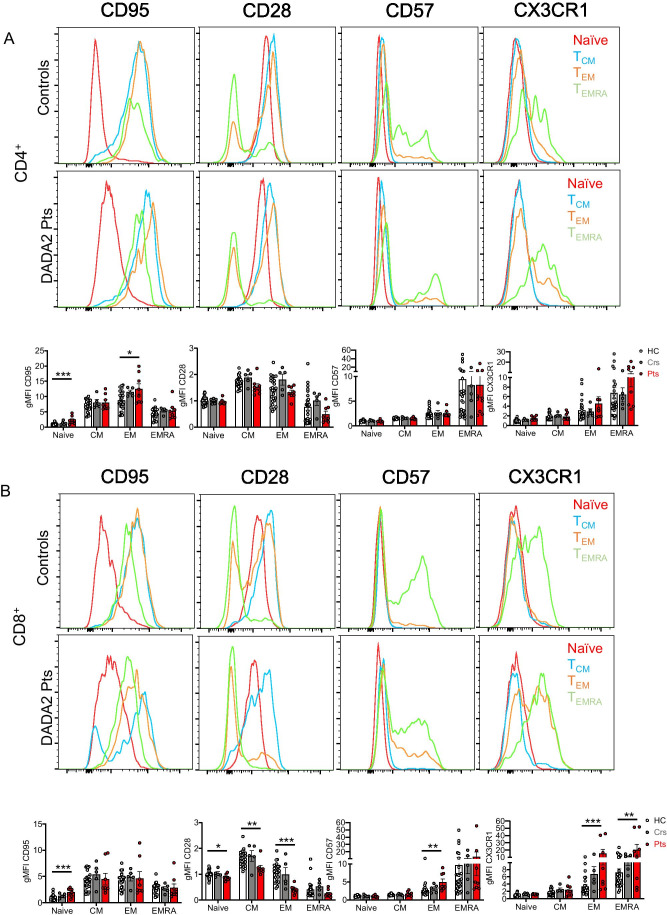
CD4^+^ and CD8^+^ T cells in DADA2 patients display an exacerbated senescent/exhausted phenotype. Expression of CD95, CD28, CD57 and CX3CR1 on naïve (red), CM (blue), EM (orange), and EMRA (green) subsets of **A** CD4^+^ and **B** CD8^+^ T cells from healthy donors, heterozygous carriers and DADA2 patients. Data are depicted as histogram plots for healthy controls and DADA2 patients (top panels) and summarized in graphs (bottom panels) which as gMFI normalized to the naive CD4^+^ (**A**) or CD8^+^ (**B**) T cells from healthy controls. Graphs represent the mean ± S.E.M.; each symbol represents an individual. Significant differences were determined by multiple *t*-tests with *P < 0.05; **P < 0.01; ***P < 0.001, indicating statistical significance

The expression of the surface receptors CD28, CD95, CD57 and CX3CR1, which change during differentiation of naïve CD4^+^ and CD8^+^ T cells to T_EMRA_ subsets, was examined to determine whether ADA2-deficient T cells exhibited features of premature senescence or exhaustion [39, 40]. CD28 is constitutively expressed by naïve CD4^+^ and CD8^+^ T cells, slightly increased on T_CM_ cells and then downregulated on T_EM_ and T_EMRA_ cells from healthy donors (Fig. 4A, B). In contrast, CD95, CD57 and CX3CR1 are absent from naïve T cells, and then incrementally increased as CD4^+^ and CD8^+^ T cells differentiate (Fig. 4A, B) [40, 41]. Compared to healthy donors, DADA2 patients exhibited significantly increased expression of CD95 on naïve CD4^+^ and CD8^+^ T cells (Fig. 4A, B). CD28 expression was lower on naïve CD8^+^ T cells in DADA2 patients and then further and significantly diminished on ADA2-deficient CD8^+^ T_CM_, T_EM_ and T_EMRA_ cells compared to healthy donors (Fig. 4B). Within the memory T cell subsets, we observed significantly elevated levels of CD95 on CD4^+^ T_EM_ cells, CD57 on CD8^+^ T_EM_ cells and CX3CR1 on CD8^+^ T_EM_ and T_EMRA_ cells (Fig. 4A, B). Thus, ADA2-deficiency exacerbates T cell differentiation, resulting in greater proportions of exhausted/senescent CD4^+^ and CD8^+^ T cells.

### ADA2 Deficiency Impairs Granzyme Production by CD8^+^ T_EM_ and T_EMRA_ Cells

**Fig. 5:**
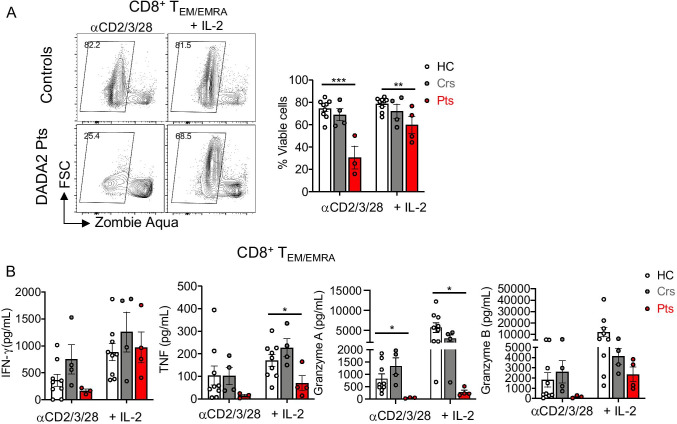
Reduced viability and impaired cytokine secretion by ADA2-deficient CD8^+^ T_EM/EMRA_ cells. Sort-purified combined T_EM/EMRA_ CD8^+^ T cells from PBMCs of healthy controls, heterozygous carriers and DADA2 patients were stimulated in vitro with anti-CD2/CD3/CD28 mAbs beads ± IL-2 for 4 days and were analyzed for viability and secretion of cytokines and cytotoxic granules. **A** Representative flow cytometric plot (left) showing viable cells. The values on the contour plots represent the percentage of ZombieAqua^−^ cells, and thus viable cells, and are summarized in graphs (right). **B** Secretion of IFN-γ, TNF, granzyme A and granzyme B by T_EM/EMRA_ CD8^+^ T cells. Graphs represent the mean ± S.E.M.; each symbol represents an individual. Significant differences were determined by multiple *t*-tests with *P < 0.05; **P < 0.01; ***P < 0.001, indicating statistical significance

To correlate alterations in surface phenotype with cell function, we sorted CD8^+^ T_EM/_T_EMRA_ cells from healthy donors, heterozygous carriers and DADA2 patients and examined survival and secretion of different cytokines after in vitro stimulation with anti-CD2/CD3/CD28 mAbs alone or together with IL-2 for 4 days. The viability of cultured ADA2-deficient CD8^+^ T_EM_/T_EMRA_ cells was significantly reduced compared to healthy donors (Fig. 5A). While exogenous IL-2 improved recovery of CD8^+^ T cells from DADA2 patients, the overall survival of these cells continued to be significantly less than that of CD8^+^ T cells from healthy donors (Fig. 5A).

The production of IFN-γ and TNF-α by ADA2-deficient CD8^+^ T_EM_ and T_EMRA_ cells was intact following in vitro stimulation. However, ADA2-deficient CD8^+^ T_EM_/T_EMRA_ cells secreted significantly lower levels of granzyme A compared to CD8^+^ T cells from healthy donors (Fig. 5B). Granzyme B secretion by ADA2-deficient CD8^+^ T cells tended to be reduced but did not reach statistical significance (Fig. 5B). CD8^+^ T_EM_ and T_EMRA_ cells from heterozygous carriers produced similar levels of cytokines and granzymes as CD8^+^ T cells from healthy donors (Fig. 5B). Reduced granzyme A secretion by activated T_EM_ and T_EMRA_ CD8^+^ T cells from DADA2 patients, combined with poor viability of and increased CD57 expression by these cells, is consistent with ADA2-deficient CD8^+^ T cells undergoing premature exhaustion and/or senescence. Unlike CD8^+^ T cells, survival, cytokine secretion and effector function of CD4^+^ T cells were unaffected by ADA2 deficiency (Fig. S3).

### Unconventional T Cells and NK Cell Subsets Are Severely Reduced in DADA2 Patients

**Fig. 6:**
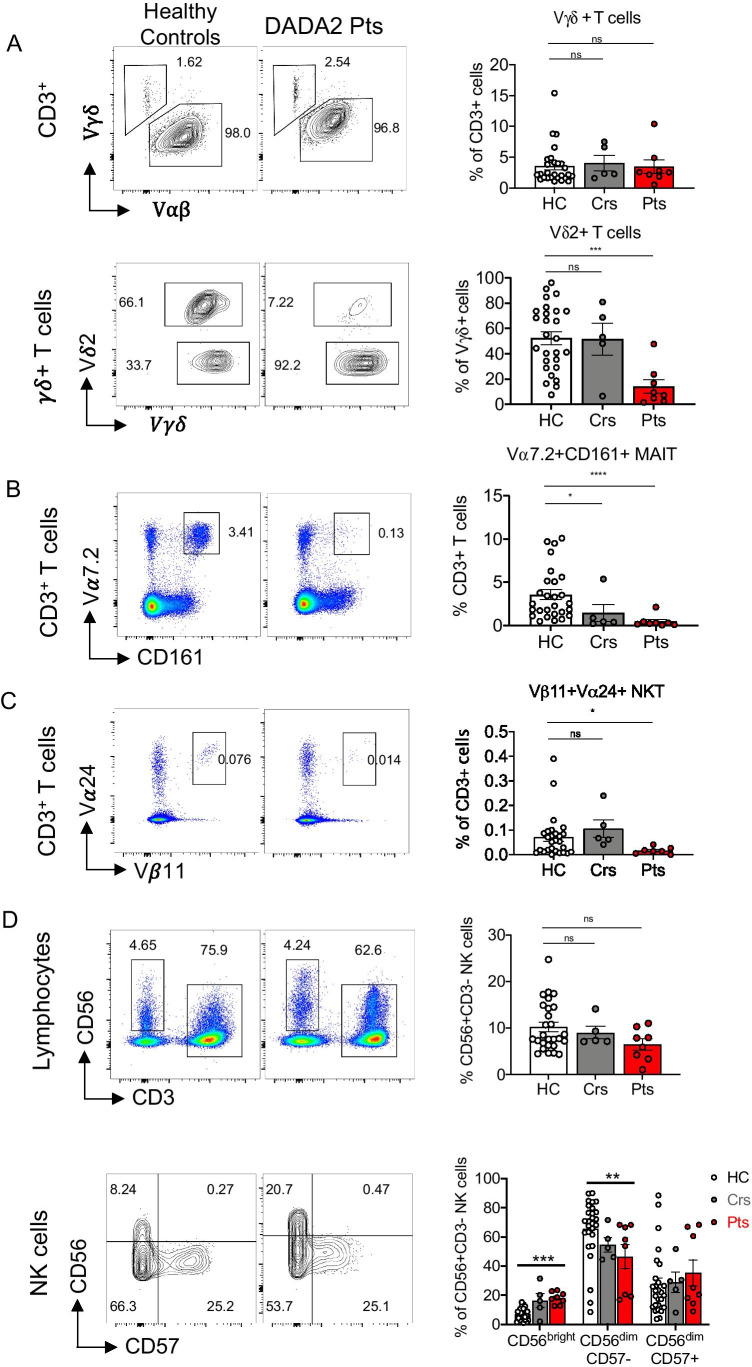
Diminished unconventional T cells and NK cell subsets in DADA2 patients. Proportions of **A** γδ^+^ T cells (top) and Vδ2^+^ within γδ^+^ T cells (bottom), **B** Vα7.2^+^CD161^+^ MAIT cells, and **C** Vβ11^+^Vα24^+^ NKT were analyzed in healthy donors, heterozygous carriers and DADA2 patients, presented as flow cytometry plots (A-C left) and summarized in graphs (A–C right). **D** representative flow cytometry plots showing total CD56^+^CD3^−^ NK cells as a proportion of lymphocytes (top), and proportions of immature CD56^bright^, mature CD56^dim^CD57^−^ and terminally differentiated CD57^dim^CD57^+^ NK cell subsets within the total NK cell population (bottom). Graphs represent the mean ± S.E.M.; each symbol represents an individual. Significant differences were determined by Mann–Whitney t-tests with *P < 0.05; **P < 0.01; ***P < 0.001; ****P < 0.0001. **A**–**D** or multiple *t-*tests with *P* < 0.05 indicating statistical significance (**D** bottom)

The consequences of ADA2 deficiency on unconventional T cells have not been investigated. Thus, we quantified proportions of γδ^+^ T cells, Vα7.2^+^CD161^+^ mucosal-associated invariant T (MAIT) cells and Vβ11^+^Vα24^+^ invariant natural killer T (iNKT) cells within CD3^+^ T cells. The proportions of γδ^+^ T cells were similar in healthy donors, heterozygous carriers and DADA2 patients (Fig. 6A top). However, within the γδ^+^ T cell subset, DADA2 patients had significantly fewer Vδ2^+^ cells than healthy donors (Fig. 6A bottom). MAIT cells (Fig. 6B) and iNKT cells (Fig. 6C) were also drastically (5–10-fold) and significantly reduced in DADA2 patients compared to healthy donors.

Within the lymphocyte population, NK cells were present in comparable proportions in healthy controls, heterozygous carriers and DADA2 patients (Fig. 6D, top). However, when we quantified distinct NK cell subsets [42, 43], immature CD56^bright^ NK cells were markedly increased and mature CD56^dim^CD57^−^ NK cells were significantly reduced in DADA2 patients (Fig. 6D, bottom). Thus, pathogenic *ADA2* mutations compromise the generation and/or maintenance of MAIT cell, NKT cells and Vδ2^+^ γδ^+^ T cells, as well as NK cell differentiation.

### Effect of ADA2 Deficiency on Dendritic Cells and Monocytes

**Fig. 7:**
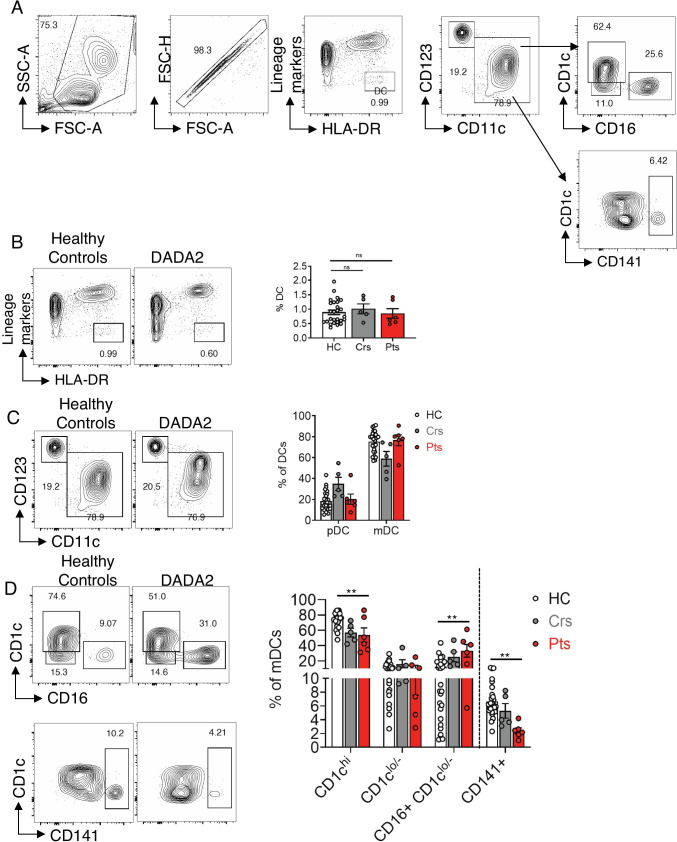
Dendritic cell subsets in ADA2 deficiency. **A** Gating strategy for DC subsets. A broad gate containing lymphocyte and monocyte populations was first gated against SSC-A vs FSC-A, followed by single cells gated as FSC-H vs FSC-A. DCs were defined as negative for lineage markers (CD3, CD19, CD20, CD56, CD14, CD235a), and HLA-DR^+^. DCs were gated as CD123 and CD11c to distinguish CD123^+^CD11c^−^ plasmacytoid DCs (pDC) and CD123^−^CD11c^+^ myeloid (conventional) DCs. mDCs were gated as CD1c and CD16 to distinguish CD1c^hi^, CD1c^lo/−^ and CD16^+^CD1c^lo/−^ mDCs and for CD1c and CD141 to identify CD141^+^ mDCs. **B–D** Representative flow cytometry plots and summary graphs showing proportions of **B** Lin^−^HLA-DR^+^ DCs, **C** pDCs and mDCs within the DC population and **D** CD1c^hi^, CD1c^lo/−^, CD16 + CD1c^lo/−^ and CD141^+^ mDC subsets. Graphs represent mean ± S.E.M.; each symbol represents an individual. Significant differences were determined by Mann–Whitney (**B**) or multiple (**C**, **D**) *t-*tests (**P < 0.01)

ADA2 is highly expressed in dendritic cells (DCs) and monocytes [44]. DCs were identified in PBMCs as HLA-DR^+^ and lineage marker (CD19, CD20, CD3, CD56, CD14, CD235a)^−^ cells (Fig. 7A). Proportions of DCs in healthy donors, heterozygous carriers and DADA2 patients were similar (Fig. 7B). Two major DC subsets can be resolved: CD123^+^CD11c^−^ plasmacytoid DCs (pDCs) and CD123^−^CD11c^+^ myeloid DCs (mDCs or conventional DCs) [45]. These subsets were detected at comparable frequencies in DADA2 patients and healthy controls (Fig. 7C). When mDC subsets were further analyzed, consistent with previous studies, we found that CD1c^hi^ mDCs were the major mDC subset in healthy donors, while CD141^+^ mDCs were least frequent (Fig. 7D) [46]. Compared to healthy donors, DADA2 patients had significantly reduced proportions of CD1c^hi^ and CD141^+^ mDCs subsets, comparable proportions of CD1c^lo/−^ mDCs and significantly increased proportions of CD16^+^CD1c^lo/− m^DC (Fig. 7D). Altogether, these data showed that while ADA2-deficiency does not impact overall numbers and proportions of dendritic cells, it does alter the distribution of mDC subsets.

**Fig. 8:**
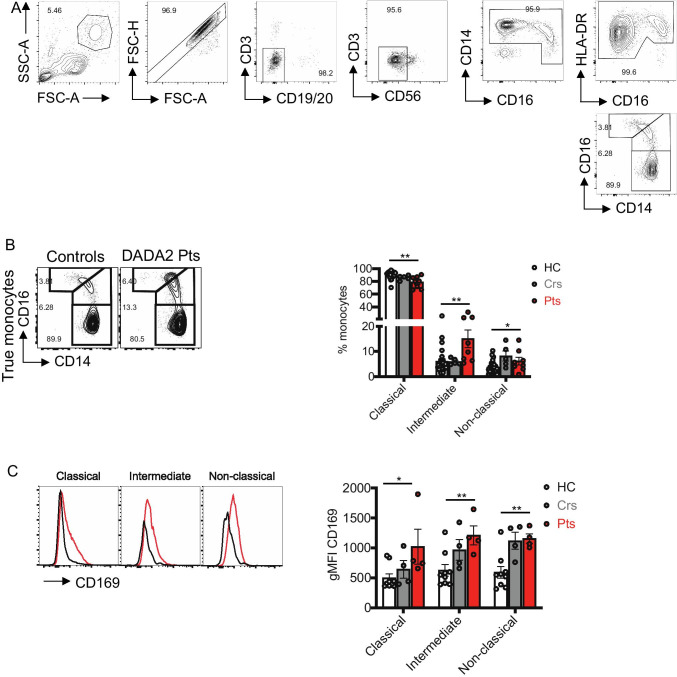
Classical monocyte are reduced and IFN-signature detected in ADA2 deficiency. **A** Gating strategy for monocyte subsets. A broad monocyte gate was set according to SSC-A vs FSC-A, then single cells defined by FSC-H by FSC-A. Lineage markers CD3, CD19/CD20 and CD56 were used to exclude T, B and NK cells. CD14^−^CD16^−^ cells were also excluded. HLA-DR^int/+^ cells that were CD16^−^ or CD16^+^ were defined as “true” monocytes. Finally, three subsets were defined: CD14^+^CD16^−^ classical, CD14^+^CD16^+^ intermediate and CD14^−^CD16^+^ non-classical monocytes (23). **B** Flow cytometry plots (left) showing the frequencies of classical (CD14^+^CD16^−^), intermediate (CD14^+^CD16^+^) and non-classical (CD14^−^CD16^+^) monocytes and summarized in graphs (right). **C** Histograms showing CD169 expression on classical, intermediate and non-classical monocytes, and gMFI of CD169 summarized in graphs (right). Significant differences were determined by multiple *t-*tests (*P < 0.05; **P < 0.01)

The predominant monocyte subset in healthy donors, heterozygous controls and DADA2 patients is the classical (CD14^+^CD16^−^) subset, followed by intermediate (CD14^+^CD16^+^) and then non-classical (CD14^−^CD16^+^) subsets (Fig. 8A, B). Compared to healthy donors, proportions of classical monocytes were significantly reduced in DADA2 patients, with a concomitant and significant increase in intermediate and non-classical monocytes (Fig. 8B). The increase in non-classical monocytes was also significant in the carrier group (Fig. 8B).

Several studies have reported a predominant type I IFN signature, in some papers in combination with a neutrophil signature, or type II IFN signature, in DADA2 patients [47–50]. We here also report increased type I IFN signature in all patients, but not in carriers, using a 6-gene signature as previously described [33] (Table 1). For this reason, we analyzed the expression of CD169/SIGLEC1, a cell adhesion protein induced by type I IFN-signaling and a potential myeloid cell-specific biomarker for an overt type I IFN response [51] on monocytes. While CD169/SIGLEC1 is expressed at similar levels on classical, intermediate and non-classical monocyte subsets present in healthy donors, its level of expression was found to be twofold higher on all subsets of ADA2-deficient monocytes (Fig. 8C). Thus, ADA2-deficiency not only alters the distribution of myeloid cell subsets but also modulates the phenotype of monocytes in terms of type I IFN dysregulation.

## Discussion

In this report, we provide extensive immunophenotyping and in vitro functional analyses of leukocytes from 10 patients with DADA2. We confirm the defect in terminal B cell differentiation in ADA2 deficiency [1, 2, 16, 52], evidenced by lower proportions of the total as well as IgG- and IgA-expressing memory B cells in these patients. Furthermore, the phenotype of ADA2-deficient B cell subsets suggested these cells were less mature than corresponding B cells in healthy donors. Moreover, the peripheral B cell defect correlated with a block in B cell development in the BM at the pre–B cell stage, as well as an intrinsic failure of DADA2 B cells to differentiate into IgA-producing B cells. As the proportions of CD27^+^ memory B cells increase in the peripheral blood of healthy individuals with age, it could be argued that the memory B cell deficiency in DADA2 reflects the younger age of some of the patients in our cohort (< 10 years old, Table 1). However, this is unlikely because frequencies and numbers of memory B cell in healthy individuals reach adult levels by 2–4 years of age [24, 53, 54]. Indeed, all but 1 patient (P7) had a paucity of memory B cells, including P8, P9 and P10 who were > 40 years old, indicating age is not a determinant of impaired B cell differentiation in DADA2. Collectively, these data demonstrating an intrinsic maturation B cell defect due to ADA2-deficiency may explain the hypogammaglobulinemia and B cell lymphopenia characteristic of DADA2 patients. Moreover, there was no improvement in hypogammaglobulinemia despite treatment with etanercept (Table 1, data not shown) [12]. This may reflect the requirement for TNF in establishing and maintaining the anatomical structure of B-cell follicles in mice [55], and/or the impact of TNF blockade on memory B cell maintenance in secondary lymphoid tissues [56].

Extended lymphocyte immunophenotyping and functional analysis also revealed several anomalies in DADA2 patients. First, we found a significantly reduced proportion of Treg cells. This has also been noted previously in some [13], but not all [16] studies. This is interesting as patients with DADA2 experience multiple auto-immune features including cytopenia and auto-antibody production [3]. Moreover, the frequencies of naïve CD4^+^ and CD8^+^ T cells were increased while T_CM_ and T_EM_ cells were reduced, pointing to a T cell differentiation defect. Second, ADA2-deficient CD4^+^ and CD8^+^ memory T cells exhibited a dysfunctional phenotype—elevated expression of PD-1, CD95, CX3CR1 and CD57; diminished expression of CD28—that has been associated with poor proliferation, terminal differentiation and exhaustion/senescence [57]. Third, ADA2-deficient memory CD8^+^ T cells exhibited diminished survival and granzyme production in vitro. Fourth, DADA2 patients were also found to have significantly lower proportions of MAIT, NKT, Vγδ2^+^ T cells and mature CD56^dim^CD57^−^ NK cells, but increased proportions of immature CD56^bright^ NK cells. Interestingly, Vγδ2 T cells are the most abundant γδT cells in peripheral blood of healthy individuals and display increased cytotoxicity against multiple viral and tumoral antigens [58, 59]. Indeed, an inverse correlation between proportions of Vδ2^+^ T cells and EBV reactivation has been observed following HSCT [60, 61].

Taken together, it is likely that the senescent/exhausted phenotype and impaired production of cytolytic molecules by ADA2-deficient CD8^+^ T cells, coupled with decreased frequencies of Vγδ2^+^ T cells and an accumulation of less mature NK cells, contribute to refractory/recurrent viral infections in ADA2-deficient patients [62–64]. From a mechanistic perspective, ADA2 deficiency may lead to increased activation of adenosine 2A receptors, reducing T cell activation, in addition to reduced TCR signaling and lytic activity of cytototoxic lymphocytes [65]. These findings are reminiscent of our previous analysis of the CD8^ +^ T cell compartment in individuals with pathogenic bi-allelic inactivating variants in *DOCK8* [40, 66, 67] or gain-of-function variants in *PIK3CD* [39] who are also susceptible to recurrent infections with herpes viruses and have an abundance of dysfunctional exhausted-type CD8^+^ T cells. The defect in ADA2-deficient cytotoxic lymphocytes may also explain why some DADA2 patients present with hemophagocytic lymphohistiocytosis (unpublished observation) or CD3^+^CD8^+^ large granular lymphocytes [68, 69].

Interestingly, DC phenotyping showed significantly increased proportions of CD16^+^CD1c^lo/−^ DCs. These DCs or non-classical like monocytes have potent T cell stimulatory capacity and produce proinflammatory cytokines upon TLR stimulation by TNF-α, IL6, IL12. Thus, these cells may play a role in maintaining the inflammatory state observed in DADA2. In addition, reduced proportions of mDCs may be associated with the overall defect in T and B cell immune responses observed in vivo.

DADA2 patients exhibited significantly reduced proportions of classical monocytes and increased proportions of intermediated and non-classical monocytes. Moreover, CD169/SIGLEC1 expression on each monocyte subset in DADA2 patients was significantly elevated compared to healthy controls. The increased non-classical monocytes in DADA2 may account in part for the observed inflammation in these patients. Indeed, nonclassical monocytes are considered proinflammatory and a prominent source of TNF-α compared to classical monocytes [70, 71]. Increased CD169/SIGLEC1 expression is in line with reported evidence of upregulated type I IFN signaling in DADA2. The concomitant increase in proportions of intermediate monocytes is interesting, as increases in both intermediate and nonclassical monocyte subsets have been observed in patients with lupus and sepsis, which are also characterized by type 1 IFN signatures [72]. Intriguingly, several patients with DADA2 were initially diagnosed with lupus based on clinical features and serum anti-dsDNA auto-antibodies [1, 11]. Interestingly, increased expression of SIGLEC-1 on myeloid cells has been implicated in viral dissemination in models of HSV1 and CMV infections, among other viruses [73]. Further study of the effect of increased SIGLEC-1 expression on the observed viral infections in DADA2 may shed light on the intriguing coalescence of the type I IFN signature and susceptibility to viral infection. Finally, increased SIGLEC-1 expression on monocytes from heterozygous carriers is consistent with the clinical observation that some carriers of pathogenic *ADA2* mutations exhibit signs and symptoms compatible with DADA2, although the carriers tested here were reportedly asymptomatic. This biological parallel of clinical phenotype in carriers is also evident from the investigation of ADA2 deficient neutrophils [74].

**Fig. 9:**
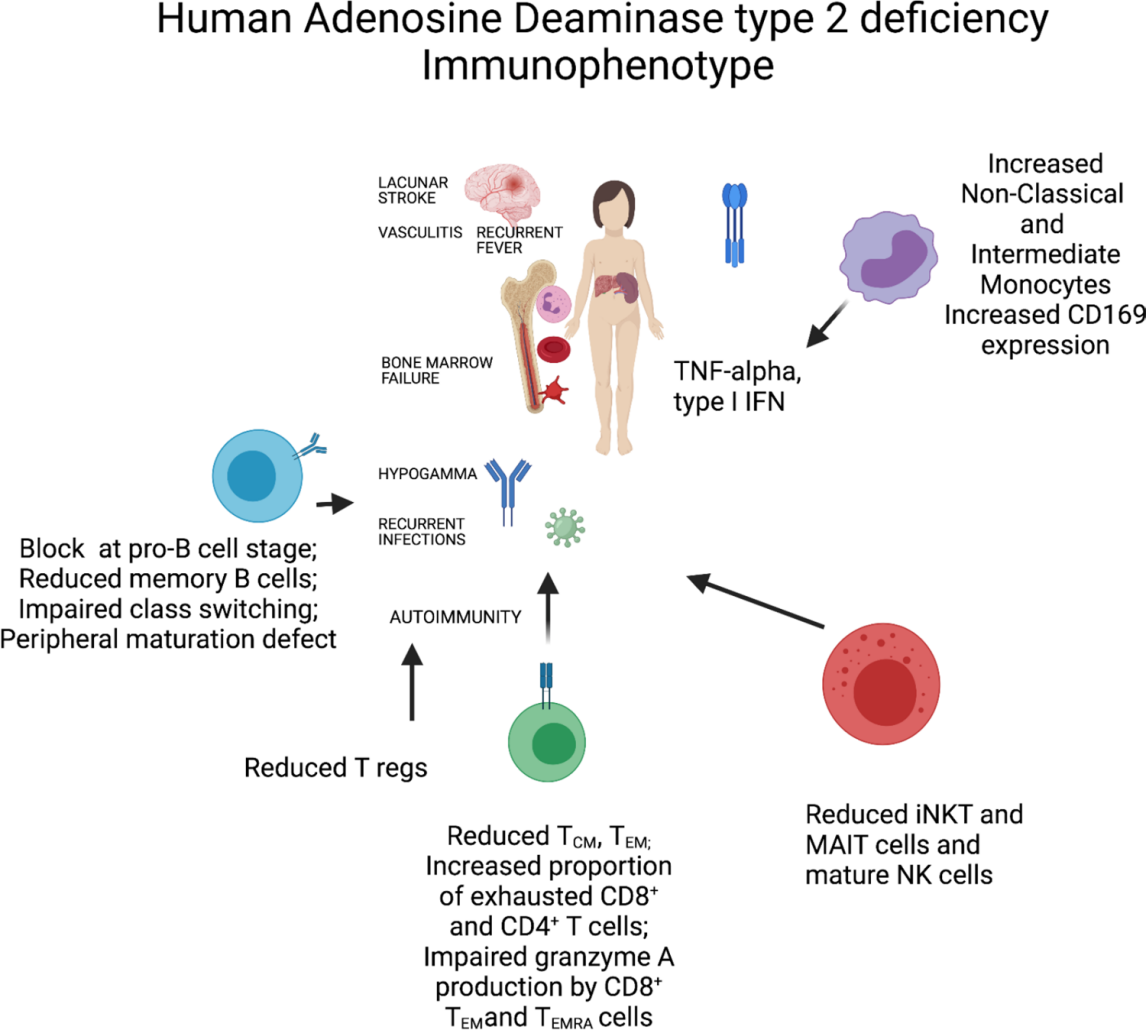
Graphical overview of major immunophenotypic findings in ADA2 deficiency. Arrows depict a hypothetical link of the immunophenotype with a clinical presentation (drafted in BioRender)

In summary, we have identified a complex immunophenotype in DADA2 with impaired differentiation of CD4^+^ and CD8^+^ T cells and B cells, in addition to an exhausted/senescent CD8^+^ T cell phenotype. Unconventional T cells are diminished whereas pro-inflammatory monocytes and CD56^bright^ immature NK cells are increased (Fig. 9). These findings suggest a delicate balance between type I and type II IFN as well as TNF-α-driven hyperinflammation next to a predisposition to severe viral infection and diminished antibody responses. Our findings confirm and extend a recently published report [75, 76]. Although these are associations, they invite a large prospective survey on the immunophenotype of ADA2-deficient patients to further our understanding of the variable immunodeficiency manifestations. Moreover, as DADA2 may underlie humoral immunodeficiency, patients with an immunophenotype in line with these finding could be selected to have ADA2 enzyme activity tested [12]. Our study is unique as several patients were not on any treatment. Also, the median age of the patients was young, which may lead us to believe that there is less environmental influence on the findings, in terms of longer ongoing inflammation and multiple treatments. Ultimately, better knowledge may aid in designing targeted therapy to prevent viral infections in these patients with excessive inflammation as the overarching phenotype.

## Supplementary Information

Below is the link to the electronic supplementary material.

Supplementary file1 (PPTX 1000 KB)

## Data Availability

Available upon request to the corresponding authors.
