# Supplementary material for: Intrinsic Defects in B Cell Development and Differentiation, T Cell Exhaustion and Altered Unconventional T Cell Generation Characterize Human Adenosine Deaminase Type 2 Deficiency
Source: J Clin Immunol. 2021 Oct 17;41(8):1915–35. doi: 10.1007/s10875-021-01141-0 (PMC8604888; doi:10.1007/s10875-021-01141-0)
Supplement: Supplementary file 1 — Supplementary file1 (PPTX 1000 KB) [file 10875_2021_1141_MOESM1_ESM.pptx]

## Slide 1
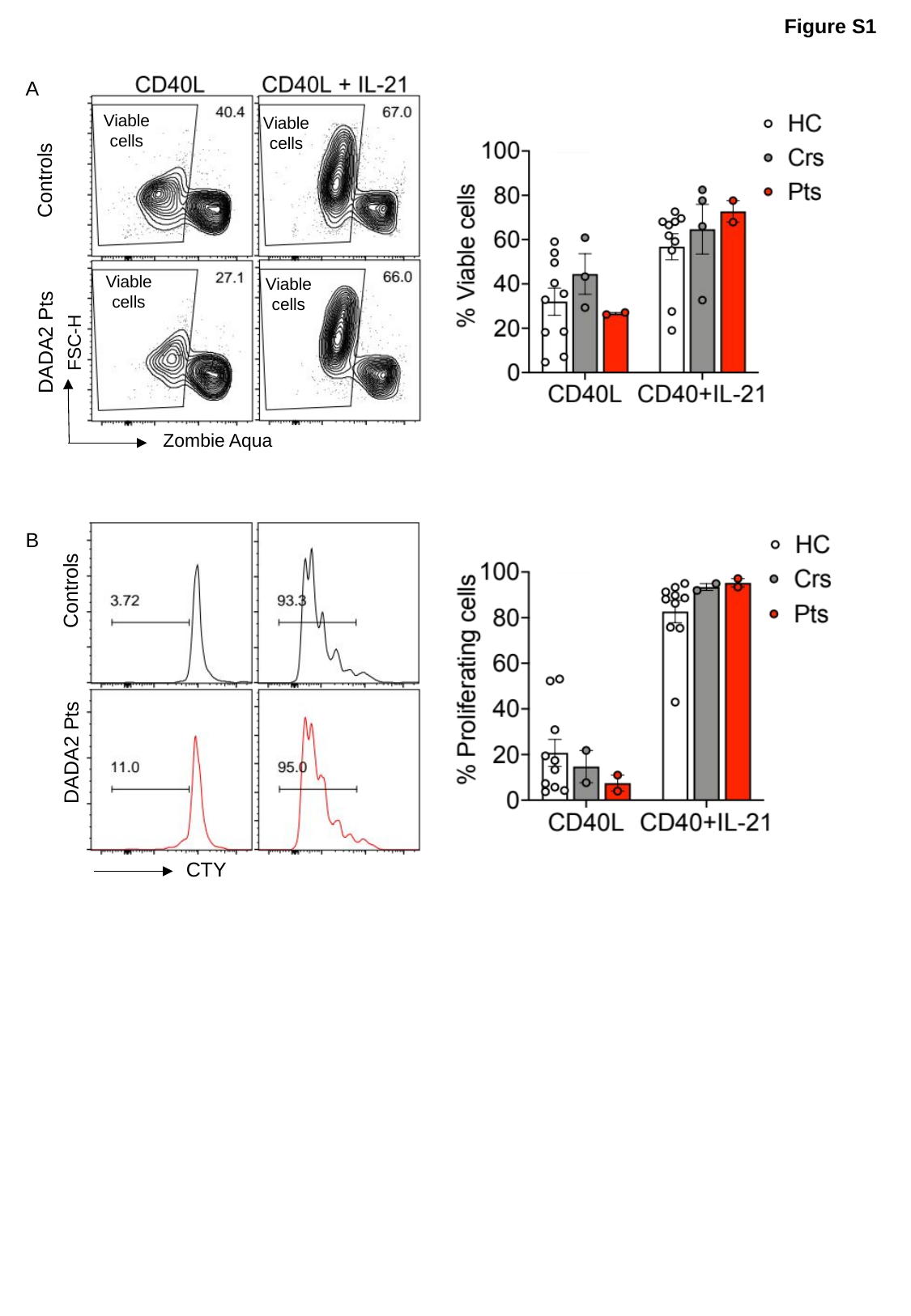

Figure S1
A
Viable cells
Viable cells
Controls
Viable cells
Viable cells
FSC-H
Zombie Aqua
DADA2 Pts
B
Controls
DADA2 Pts
CTY

## Slide 2
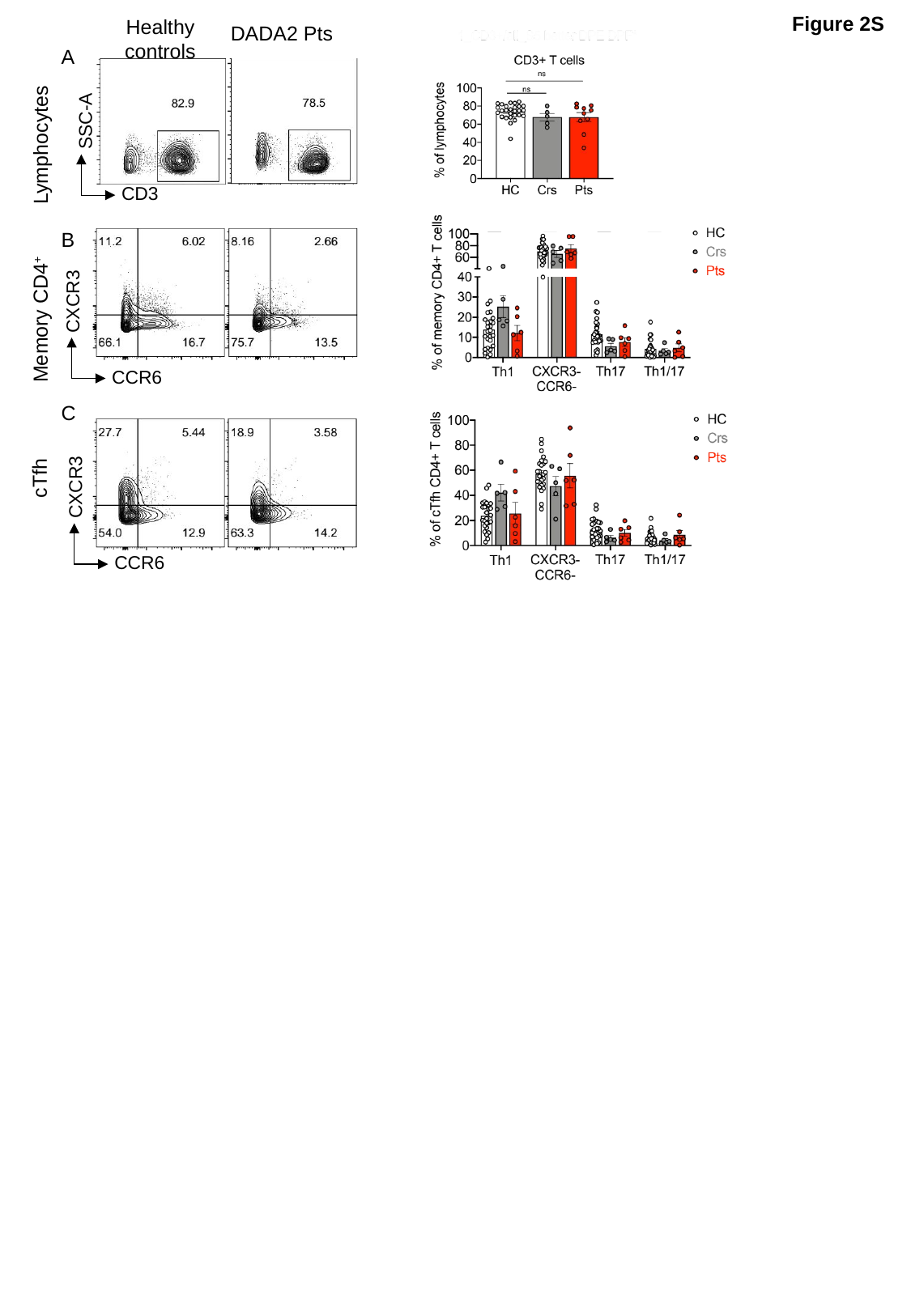

Figure 2S
Healthy controls
DADA2 Pts
A
SSC-A
CD3
Lymphocytes
B
CXCR3
CCR6
Memory CD4+
C
CXCR3
CCR6
cTfh

## Slide 3
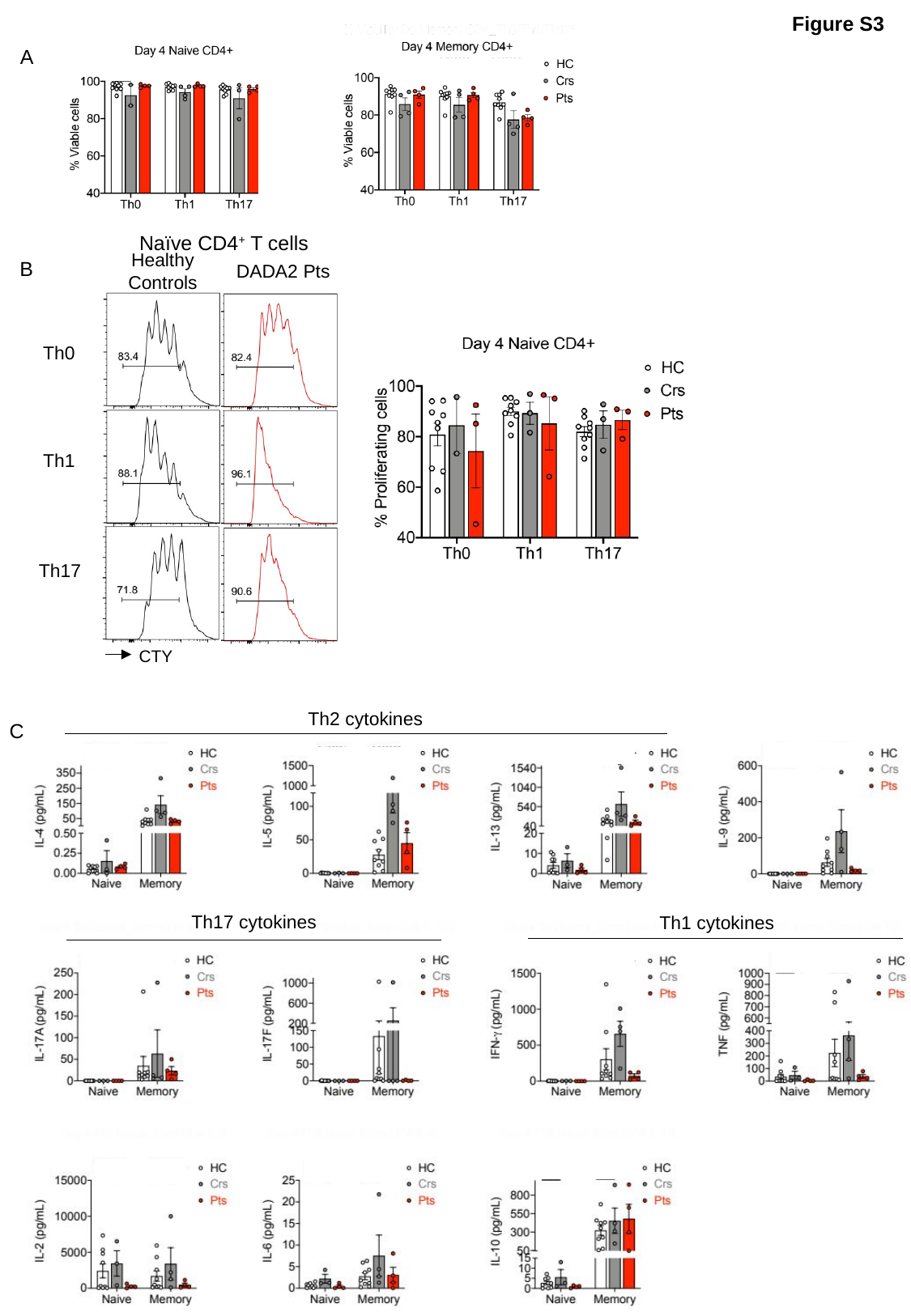

Figure S3
A
≈
Naïve CD4+ T cells
Healthy Controls
B
DADA2 Pts
Th0
Th1
Th17
CTY
Th2 cytokines
C
Th17 cytokines
Th1 cytokines
